# Novel transparent patch as an adjunct to adult pulmonary valve replacement

**DOI:** 10.1007/s11748-025-02154-x

**Published:** 2025-05-11

**Authors:** Hajime Ichikawa, Shigemitsu Iwai, Yasumi Nishiwaki, Kousuke Kikuchi

**Affiliations:** 1 Department of Pediatric Cardiovascular Surgery, National Cerebral and Cardiovascular Center, 6-1 Kishibe-Shimmachi, Suita, Osaka 564-8565 Japan; 2 Implantable Medical Device Development Department, Teijin Limited., Kasumigaseki Common Gate West Tower, 2-1, Kasumigaseki 3-chome, Chiyoda-ku, Tokyo, 100-8585 Japan; 3 Cardiovascular Surgery, Japan Community Health Care Organization Osaka Hospital, 4-2-78 Fukushima, Fukushima-ku, Osaka, 553-0003 Japan

**Keywords:** Adult congenital heart disease, Biocompatible biodegradable polymer, Pulmonary valve replacement, Tissue regeneration, Transparent material

## Abstract

**Objective:**

Patients with congenital heart defects, such as tetralogy of Fallot (TOF) or right ventricular outflow tract stenosis or atresia, often require pulmonary valve replacement (PVR) decades after the primary repair. The purpose of this study was to assess the safety and efficacy of a novel synthetic hybrid fabric (SHF) for PVR in adult congenital heart disease.

**Methods:**

SHF, consisting of bio-absorbable and non-absorbable yarns coated with cross-linked gelatin, was used in a prospective, multicenter, single-arm pivotal clinical trial involving subjects with an age range of 0–59 years. The overall study was registered in the Japan Registry of Clinical Trials (jRCT1080224691). This paper specifically presents a subgroup analysis focusing on five adult patients (aged 18–42 years) from the multicenter trial.

**Results:**

The procedures were performed similarly to those using existing products, with no SHF-specific complications observed. The SHF material allowed surgeons to clearly observe the bioprosthetic valve annulus during suturing. None of the patients required blood transfusion or developed adverse events. At a mean follow-up of 4.5 years (range 4.0–4.9 years), no re-interventions or reoperations were needed.

**Conclusion:**

SHF shows promise as a patch material for PVR, offering significant benefits such as clear visualization during surgery, which facilitates precise valve placement. This transparency is crucial for adults with repaired TOF, as it helps reduce surgery time and complication risks. This study suggests that SHF could be a valuable material for adult PVR, extending its potential applications beyond pediatric cardiology.

**Supplementary Information:**

The online version contains supplementary material available at 10.1007/s11748-025-02154-x.

## Introduction

The number of transcatheter pulmonary valve replacements has been increasing recently. However, surgical pulmonary valve replacement (PVR) still has an important role in the treatment of “repaired” adult congenital heart disease (ACHD) patients with right ventricular outflow tract (RVOT) dysfunction. In such cases, the valve is typically sutured to native tissue on the posterior wall of the RV outflow, and an appropriately sized patch is inserted to cover the anterior wall and provide sufficient space for the bioprosthetic valve [1].

Throughout the history of PVR, clinicians have relied on various materials—artificial vascular grafts, ePTFE patches, and xenograft pericardial patches—for reconstructive procedures in the RVOT. Given the increased propensity for calcification in xenograft pericardial patches, a considerable number of healthcare institutions in Japan have transitioned to ePTFE patches or artificial vascular grafts. However, these materials are inherently foreign to the body and pose complications for potential re-intervention. More recently, the emergence of transcatheter pulmonary valve replacement techniques has offered a minimally invasive option for adults with congenital heart defects necessitating pulmonary valve replacements [2]. However, this innovative approach is contraindicated for patients with pre-existing calcified patches or grafts from prior surgeries. As a result, there is a protracted and unmet need for calcification-resistant patching materials. In addition, ACHD patients frequently present with complex surgical anatomy due to previous operations and remodeling over time [3, 4]. In such cases, poor visualization caused by opaque patch materials may complicate precise placement of the prosthetic valve and extend operative time.

Recently, a novel medical material that accommodates in situ tissue regeneration (synthetic hybrid fabric [SHF]) has been developed for use as a cardiovascular patch. SHF is a knitted fabric consisting of biodegradable synthetic polymeric yarn (poly-L-lactic acid, PLLA) and non-biodegradable synthetic polymeric yarn (polyethylene terephthalate, PET) coated with a cross-linked gelatin membrane [5].

Studies in animal models have shown successful vascular regeneration and assimilation by native vascular tissue. Compared to conventional products, SHF has been associated with a markedly lower incidence of chronic inflammation and calcification in long-term use [6, 7]. The safety and efficacy of SHF in cardiac repair surgery for congenital heart disease have been confirmed through a recently completed prospective, multicenter, single-arm pivotal clinical trial [8]. SHF has also recently obtained manufacturing and marketing approval from Japan's Pharmaceuticals and Medical Devices Agency (PMDA). It is marketed by Teijin Medical Technologies Co., Ltd. under the name SYNFOLIUM.

## Methods

### Subjects

This study represents a prospective subgroup analysis from a multicenter clinical trial (jRCT1080224691), specifically focusing on adult patients undergoing PVR using SHF. This study enrolled five patients (two men and three women) aged 18–42 years, with a history of multiple reoperations. Four patients had tetralogy of Fallot as the primary disease. RVOT angioplasty was performed in three patients, and pulmonary angioplasty in two. Approval was obtained from the Institutional Review Board at the National Cerebral and Cardiovascular Center (#1103; 2019).

### Procedure

A longitudinal incision was made proximal and distal to the pulmonary annulus. The biological valve was held by grasping the sewing cuff with forceps, without the use of a valve holder. Any residual portions of the native pulmonary valve were resected, and a porcine bioprosthetic aortic valve was used, with a valve size of 25 mm or more being preferred. Suturing began at the bottom of the valve annulus and continued along about two-thirds of the posterior circumference, using 4–0 monofilament suture.

After suturing the posterior aspect of the bioprosthesis, SHF (thickness 0.2–0.4 mm, standard dimensions 80 mm × 130 mm) was trimmed, and suture placement from the pulmonary artery aspect was initiated using 5–0 monofilament thread. After suturing up to the valve annulus, the suture thread used on the posterior aspect of the bioprosthetic valve and the suture thread used on the patch were ligated.

The anterior aspect of the sewing cuff was then sutured to SHF. It was important to pull SHF distally and angle it posteriorly to the bioprosthetic valve. The position of the suture line was carefully determined to avoid anterior tilting of the valve. Finally, SHF was sutured to the RVOT using 4–0 monofilament suture.

## Results

### Patient characteristics

Five patients (3 females, 2 males) with a history of congenital heart defects underwent PVR using SHF. The mean age at PVR was 30.8 years (range: 18–42 years). Four patients had a primary diagnosis of TOF, while one had double outlet right ventricle (DORV). All patients had undergone previous intracardiac repair with transannular patch (range: 4 months to 12 years 2 months) (Table 1).

**Table 1:** Adult PVR with synthetic hybrid fabric: patient characteristics and outcomes

Patient	1	2	3	4	5
Sex	F	F	M	F	M
Age at PVR (years)	36	42	18	20	38
Body weight (kg)	61.4	38.1	55.0	44.7	61.5
Age at intracardiac repair (transannular patch)	3 yr 2 mo	3 yr	0 yr 4 mo	3 yr 8 mo	12 yr 2 mo
Primary disease	TOF	TOF	TOF	DORV	TOF
Indication for reoperation	PI	PI	PI	PI	PI
Reoperation	PVR	PVR, TAP, Residual VSD closure	PVR TAP	PVR	PVR TAP
Patch location	RVOT patch	RVOT patch	PA trunk patch	RVOT patch	RVOT patch
Patch size (mm)	80 × 35	80 × 35	30 × 20	60 × 30	60 × 35
Diameter of bioprosthetic valve	25 mm Mosaic	25 mm Mosaic	25 mm Mosaic	25 mm Mosaic	25 mm Mosaic
Follow-up (years)	4.9	4.7	4.3	4.4	4.0
Adverse event	None	None	None	None	None
UCG findings	n.p	n.p	n.p	n.p	n.p
Flow velocity (m/s)	0.7	1.3	2.1	0.6	0.9

*PVR* pulmonary valve replacement, *TOF* tetralogy of Fallot, *DORV* double outlet right ventricle, *PI* pulmonary insufficiency, *TAP* tricuspid annuloplasty, *VSD* ventricular septal defect, *RVOT* right ventricular outflow tract, *UCG* ultrasound cardiography

### Procedural outcomes

**Fig. 1:**
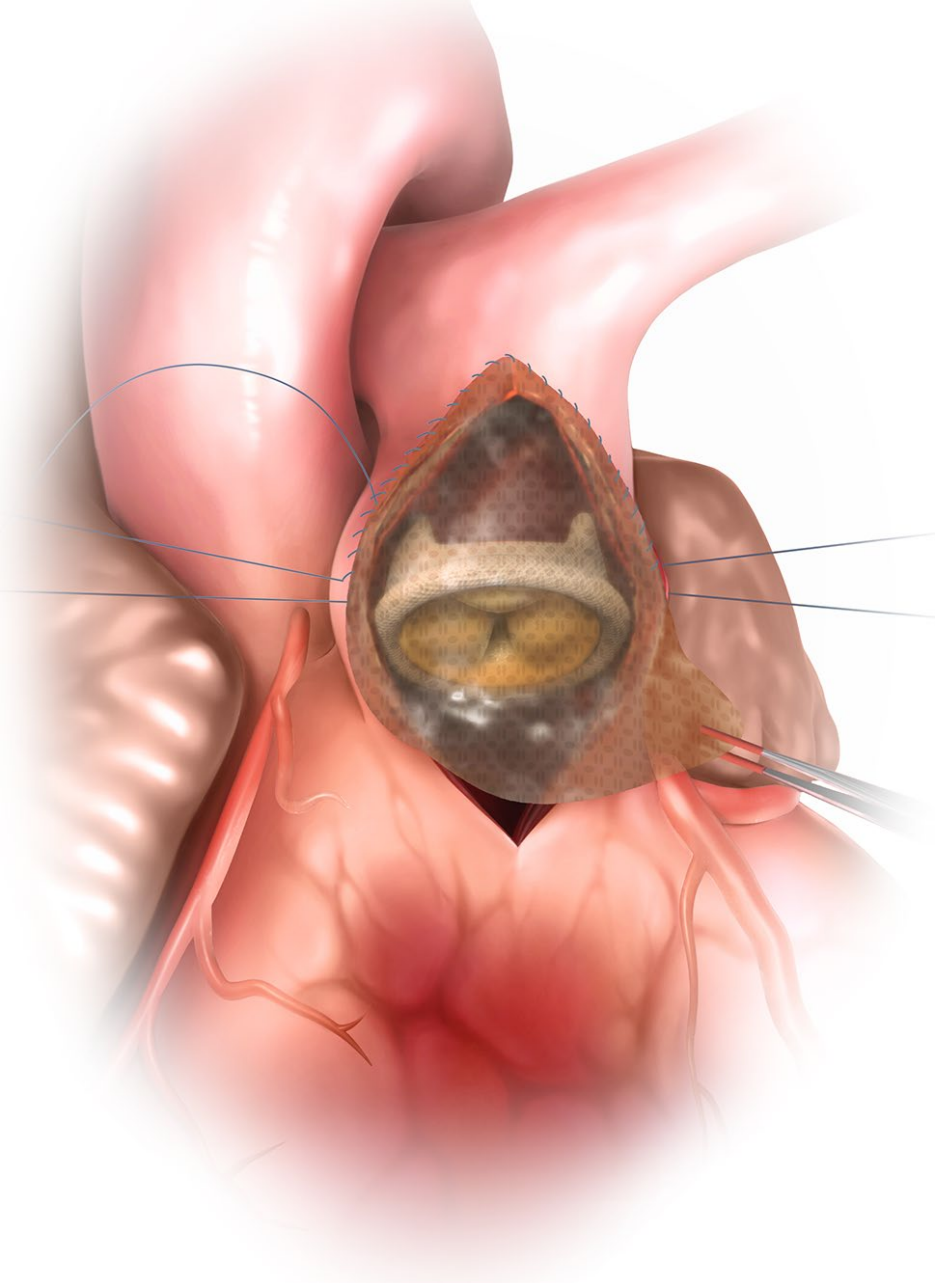
After suturing the posterior bioprosthesis, SHF (0.2–0.4 mm, 80 mm × 130 mm) was trimmed and sutured from the pulmonary artery with 5–0 monofilament thread. The threads from the valve and patch were ligated at the annulus. SHF’s transparency allowed direct visualization for precise positioning

The PVR procedure using SHF was performed similarly to those using existing products, with no SHF-specific complications observed. The transparency of SHF allowed surgeons to visually confirm the precise positioning of the bioprosthetic valve annulus during suturing (Fig. 1), facilitating faster and more precise surgery (Video 1). SHF was soft, conformed readily to the shape of the blood vessel, and was easy to fit, with less bleeding through suture holes compared to conventional products.

All patients received a 25 mm Mosaic bioprosthetic valve. SHF patches were used predominantly for RVOT reconstruction in four patients, with one patient receiving a pulmonary artery (PA) trunk patch. The size of the patches used in the study ranged from 30 × 20 mm to 80 × 35 mm.

### Follow-up and clinical outcomes

The mean follow-up duration was 4.5 years (range: 4.0–4.9 years). Patients underwent regular postoperative monitoring, including echocardiographic evaluations and clinical assessments at scheduled intervals. No adverse events were reported in any of the patients during the follow-up period.

Echocardiographic findings at the latest follow-up were unremarkable for all patients, indicating stable valve function. The flow velocity across the pulmonary valve ranged from 0.6 to 2.1 m/s, suggesting effective hemodynamic function without evidence of significant stenosis or regurgitation. Importantly, no patients required re-intervention or reoperation up to four years post-surgery.

Table 1 summarizes the specific follow-up data for each patient, including demographic information, surgical details, and key outcome measures.

## Discussion

Adults with repaired TOF, especially those with transannular patch repair, often require PVR due to severe pulmonary regurgitation, right ventricle dilation, and dysfunction. Precise positioning of the bioprosthetic valve is crucial in PVR procedures using patches.

While conventional patch materials such as ePTFE and xenograft pericardium are widely used, their opacity can limit visualization of anatomical landmarks during surgery. The lack of reported problems related to this limitation may reflect the experience and skill of congenital heart surgeons. However, ACHD patients often present with complex anatomy due to previous operations and remodeling, making surgery more technically challenging [3, 4].

In this context, transparent patches such as SHF offer intraoperative advantages by allowing direct visual confirmation of the suture line and annular position. In our series, SHF enabled more precise valve placement and was associated with smoother surgical flow. The patch’s soft texture and ease of handling were particularly beneficial in reoperative settings where tissue planes were distorted.

Although a double-patch technique (placing separate patches above and below the valve) can improve visualization, it adds procedural complexity and prolongs surgery. SHF combines structural coverage and visualization in a single patch, potentially simplifying the operation. Additionally, using a single transparent patch reduces the complexity and allows for better visibility when suturing the valve to the patch, enhancing safety and precision during the procedure.

SHF's ease of handling and compatibility with existing surgical techniques eliminates the need for special training, which is crucial as PVRs become more widespread. Although its regenerative properties are particularly valuable in pediatric patients, its transparency, biocompatibility, and handling characteristics are highly relevant in adults. Despite slower calcification rates in adults, many ACHD patients require re-intervention over a decade postoperatively. Therefore, materials with resistance to calcification and long-term durability—such as SHF, which has demonstrated resistance to calcification in preclinical models [6, 7]—are beneficial in this population, especially considering the increasing use of transcatheter therapies.

The positive outcomes observed in these five adult cases suggest that SHF could be valuable not only in pediatric cardiology, but also in adult PVR procedures, addressing the specific needs of this patient population.

### Study limitations

This study has several limitations. First, it was conducted as a single-arm observational analysis involving a small number of patients (*n* = 5), which limits generalizability. Second, it was performed at a single institution, and outcomes may reflect institution-specific practices. Third, the follow-up duration was intermediate (≤ 5 years), and long-term durability remains to be determined. Fourth, surgical handling assessments were partly subjective and not formally quantified.

Although SHF offers tissue regenerative potential applicable across age groups, growth compatibility is less relevant in adults. Nevertheless, its transparency and handling benefits were particularly advantageous in reoperative cases. Future studies with larger cohorts, longer follow-up, and multicenter comparative designs are needed to confirm these findings and further define SHF’s clinical utility in ACHD patients.

## Conclusions

This study demonstrates that SHF is a promising material for PVR in ACHD patients. The transparency of SHF facilitates precise valve placement, reducing surgery time and complication risks. The absence of adverse events and the need for re-intervention or reoperation up to 4 years post-surgery further support its efficacy and safety.

## Supplementary Information

Below is the link to the electronic supplementary material.

Supplementary file1 Video 1 Novel transparent patch was used in pulmonary valve replacement (MPG 45654 KB)

## Data Availability

The data from this study will be shared on reasonable request to the corresponding author.
